# Intraoperative floppy iris syndrome (IFIS): what complication rates can we expect?

**DOI:** 10.1007/s00417-013-2536-8

**Published:** 2013-11-27

**Authors:** Andrzej Grzybowski, Patrycja Krzyżanowska-Berkowska

**Affiliations:** 1 Department of Ophthalmology, Poznan City Hospital, ul. Szwajcarska 3, 61-285 Poznań, Poland; 2 University of Warmia and Mazury, ul.Żołnierska 14C, Olsztyn, Poland; 3 Department of Ophthalmology, Wroclaw Medical University, ul. Borowska 213, 50-556 Wroclaw, Poland

Dear Editor,

In a very interesting paper, Haridas et al. have shown that patients with intraoperative floppy iris syndrome (IFIS) receiving tamsulosin or doxazosin differ in terms of morbidity rates [1].

Among 2,785 cataract operations performed in 2,028 patients, the authors identified 52 (1.9 %) cases treated with tamsulosin and 109 (3.9 %) cases in which doxazosin was administered. They compared morbidity rates between these two groups.

In our opinion, however, some aspects of this study need to be discussed. In their retrospective analysis, the authors found at least one IFIS feature in 17 out of 106 eyes (16 %) from the doxazosin group and in 25 out of 52 eyes (48 %) from the tamsulosin group. Alpha-1 antagonists, used in the symptomatic treatment of benign prostatic hyperplasia, may increase the surgical difficulties and risk of iris damage, corneal edema, posterior or anterior capsule rupture, and postoperative increase in intraocular pressure [2–4]. Nevertheless, analysis of data expressed as percentages can be misleading and provide false information on the prevalence of IFIS and expected intraoperative morbidity rate.

In Table 1 of their paper, Haridas et al. presented data on the size of the pupil and status of the iris in the tamsulosin and doxazosin groups [1]. However, the prevalence of various abnormalities (large or small pupil, iris prolapse, etc.) was expressed as a percentage of IFIS cases in a given group, i.e. per 25 and 17 eyes in tamsulosin and doxazosin group, respectively. In our opinion, however, the prevalence should be rather expressed per the whole group administered a given agent, i.e. per 52 and 106 eyes of patients treated with tamsulosin and doxazosin, respectively. Only in this way can we estimate the potential risk of intraoperative complications in patients treated with α-1 antagonists. Furthermore, it should be noted that expressing seven cases of intraoperative complications in the tamsulosin group as 13.5 % (7 out of 52 eyes) suggests that such morbidity can be expected in patients subjected to cataract surgeries. This is not true, as intraoperative complications documented in the tamsulosin IFIS group (iris damage – 5 cases, anterior capsule rupture – 1 case, corneal edema – 1 case) corresponded to only 0.3 % of all 2,785 operated eyes, the average rate of cataract complications in non-IFIS patients. Takmaz analyzed a group of 858 operated eyes and identified intraoperative complications in 6 eyes of patients who were treated with tamsulosin [8]. However, he emphasized the lack of significant differences in the morbidity rates of tamsulosin-treated patients with and without IFIS (p = 0.245). According to Haridas et al., patients treated with tamsulosin differed significantly from the controls in terms of the morbidity rates (13.5 %, i.e. 7 out of 52 eyes vs. 3.3 %, i.e. 5 out of 150 eyes; p = 0.014) [1]. However, we postulate that statistical analyses are not justified in the case of such a large disproportion in group size. Furthermore, this analysis was not adjusted for surgeon’s experience and other intraoperative factors.

**Table 1:** Studies reporting the rate of intraoperative floppy iris syndrome (IFIS) complications

	IFIS cases/all cases	IFIS cases/α-1 antagonist group	Tamsulosin IFIS/Tamsulosin group	Complication rate (% of all cases)
Haridas et al [1]. (2013)	1,5 % 42/2,785 eyes	26,6 % 42/158	48 % 25/52 eyes	AC rupture – 1 case (0,04 %); 5 eyes with iris damage (0,2 %); 1 eye corneal oedema
Chang et al [2]. (2005)	Clinical Study 1 2 % 10/511 patients	37 % 10/27 patients	63 % 10/16 patients	PC rupture & vitreous loss- 2 cases (0,4 %)
Chang et al [2]. (2005)	Clinical Study 2 2,2 % 16/741 patients	94 % 15/16 patients	100 % 15/15 patients	AC rupture - 1 case (0,1 %);
Cheung et al [5]. (2006)	1 % 17/1689 eyes	29/8 % 17/57 eyes	80 % 8/10 eyes	No complications
Oshika et al [6]. (2007)	1,1 % 29/2,643 eyes	36,7 % 29/79 eyes	43,1 % 25/58 eyes	No data
Blouin et al [7]. (2007)	4 % 61/1268 eyes	66,3 % 61/92 eyes	64,5 % 51/79 eyes	PC rupture - 1 case (0,08 %); 3 eyes with iris damage; zonular dehiscence-1 case; retained lens fragments – 1 case; CME – 2 cases
Takmaz [8] (2007)	1,6 % 14/858 eyes	Only patients taking Tamsulosin	77,8 % 14/18 eyes	PC rupture - 1 case (0,1 %); 5 eyes with iris damage (0,6 %)
Nguyen et al [9]. (2007)	Unknown	Unknown/606 cases	Unknown/363 cases	Difficult to assess, PC rupture – 7 % of unknown number of cases
Altan-Yaycioglu et al [10]. (2009)	2,8 % 14/500 patients	66,7 % 14/21 patients	66,7 % 6/9 patients	PC rupture - 1 case (0,2 %)

*IFIS* intraoperative floppy iris syndrome, *AC* anterior capsule, *PC* posterior capsule, *CME* cystoids macular oedema

The exact data on the type and prevalence of intraoperative complications is missing in available literature. (Table 1) Chang et al. spoke to the intraoperative use of retractors in five cases but did not provide any data on the percentage of postoperative iris damage in patients with IFIS [2]. In turn, the report by Oshika et al. not only lacks any data on postoperative morbidity rates but does not provide any information about intraoperative strategies of performing cataract phacoemulsification in patients with IFIS [6].

Concluding: Although the use of α-1 antagonists, especially tamsulosin, can constitute a potential risk of intraoperative complications in cataract surgery, such complications occur sporadically, and therefore, the morbidity rates should be presented in a uniform manner in order to avoid their inadvertent overestimation.
